# Sensory Impairment and Labor Force Participation: Evidence From Pre-Retirement Age Adults in China

**DOI:** 10.1093/geroni/igaf122.4190

**Published:** 2025-12-31

**Authors:** Yuetong Toria Liu, Zhang Zhang

**Affiliations:** Johns Hopkins University, Bloomberg School of Public Health, Baltimore, Maryland, United States

## Abstract

Hearing loss (HL), vision impairment (VI), and dual sensory impairment (DSI) pose challenges to labor force participation (LFP) in midlife. This study examines the impact of HL, VI, and DSI on LFP among adults aged 50-64 and explores differences by hukou-residency status in China. The data were drawn from the China Health and Retirement Longitudinal Study (2011–2018). We conducted analyses using ordinary least squares, random-effects, and fixed-effects (FE) models. FE models were applied for subgroup analyses and interpretation. Among 32,408 person-year observations, 15.4% reported HL, 6.8% reported VI, and 4.6% reported DSI. The weighted prevalence of hearing aid use among people with HL was 1.9%. No significant difference was found in the probability of employment between those with and without sensory impairment, nor across hukou-residency groups. However, HL, VI, and DSI were associated with 12.9% (p < .01), 5.5% (p > .05), and 21.3% (p < .01) fewer annual work hours, respectively, compared to those without sensory impairment. Among rural residents, HL, VI, and DSI corresponded to 16.9% (p < .01), 6.6% (p > .05), and 22.2% (p < .01) reductions in annual work hours, respectively. For rural-to-urban migrant workers, reductions were 0.9%, 6.0%, and 23.8%, respectively, although not statistically significant. Our study suggests that while sensory impairment was not associated with the probability of employment, HL and DSI significantly reduce annual work hours, particularly among rural residents. These findings point to the need for more inclusive health and labor policies to support pre-retirement adults with sensory impairments in China.

